# Maternal and fetal risk factors for stillbirth in Otjozondjupa Region, Namibia: a case-control study

**DOI:** 10.11604/pamj.2023.44.73.26724

**Published:** 2023-02-07

**Authors:** Rebekka Ndatolewe Shikesho, Taimi Amakali-Nauiseb, Kofi Mensah Nyarko

**Affiliations:** 1 University of Namibia, Faculty of Health Sciences, Windhoek, Namibia,; 2 Field Epidemiology and Laboratory Training Program (FELTP), Windhoek, Namibia

**Keywords:** Fetal death, stillbirth, determinants, risk factors

## Abstract

Introduction: stillbirth is defined as a baby born with no signs of life. Globally, around 3.2 million stillbirths occur annually, of which, 98% are experienced in low and middle-income countries. Otjozondjupa Region topped the list of regions with high burden of stillbirth in Namibia in 2016. This study sought to elucida**te risk factors for stillbirth**.

Methods: an unmatched 1:2 case-control study was conducted. A sample of 285, 95 cases and 190 controls were chosen using simple random sampling method. Bivariate and multivariate analyses were done to assess the risk factors of stillbirth.

Results: maternal medical and obstetric factors significantly associated with stillbirth are: premature delivery (aOR 0.13 95% CI 0.05, 0.33, P < 0.001), gestational age (aOR 0.04, 95% CI 0.00, 0.25, P < 0.001), high-risk pregnancy (aOR 3.59, 95% CI 1.35, 9.55, P = 0.01), duration of labor (aOR 4.04, 95% CI 1.56, 10.43, P = 0.003) and antenatal care (ANC) attendance (aOR 0.07, 95% CI 0.00, 0.79, P = 0.03). Only low birth weight (≤ 2500 g) was associated with stillbirth amongst fetal related factors (aOR 16.58, 95% CI 8.71, 31.55, P < 0.001).

Conclusion: this study concludes that stillbirth in Otjozondjupa Region was mostly associated with maternal medical and obstetric factors. It also concluded that attending antenatal care in Otjozondjupa did not improve birth outcome.

## Introduction

Stillbirth is a distressing outcome of pregnancy that needs to be evaded at all costs. It is not only a traumatic experience for the mother but for the family as well as the obstetrician [1]. The centers for disease control simply defined stillbirth as the death of a baby before or during delivery [2]. To date, there is no unanimously accepted definition of stillbirth that includes the criteria for gestational age or birth weight at which loss of life occurs [3]. The socio-economic status of the country and presence of neonatal intensive care plays an important role in the determination of the 'cut off weeks' of the gestational age for stillbirth. Fewer babies below 30 weeks gestational age survives in countries without neonatal intensive care hence the cut off gestational weeks are much lower in high income countries (HICs) as compared to middle income countries (MICs) and low income countries (LICs) [4,5]. Due to various stillbirth definitions, the World Health Organization (WHO) recommends, 28 gestational weeks and or 1000 grams or having at least 35 cm body length [6]. This is therefore the definition that was employed for this study.

In 2015 alone, an estimated 2.5 million stillbirths experienced globally were third trimester stillbirths [7]. Whilst the numbers are overwhelming, it is exhilarating to note that 98% of these occur in low and middle income countries (LMICs). Some studies have argued that rates of perinatal and neonatal mortality have decreased much slower than infant mortality rates in the 2000s [8,9]. By 2015, global stillbirth rate had decreased by 25.5% from 24.7 stillbirths per 1000 deliveries in 2000 to 18.4 stillbirths per 1000 deliveries in 2015 [10,11]. This accounted from an annual reduction rate (ARR) of 1.7% [11]. This reduction remains much slower than that of maternal mortality rate, which achieved an ARR of 3.0% and under 5 mortalities (ARR 3.9%) for the same period [10]. Taking these findings into consideration, it is evident that no significant improvement to curb stillbirths has been achieved. Potential causes of stillbirth alluded to include, high parity, lack of antenatal care (ANC), infection such as syphilis, HIV/AIDS and malaria, birth trauma, prematurity and placental problems to mention but a few [12].

Namibia is not spared from the high burden of this misfortune. Lawn *et al*. [7] pointed out that by 2015, Namibia´s stillbirth ARR merely ranged between 1.5% to > 2.0%. Stillbirths are not targeted for in the millennium development goals (MDG) and are missing in the sustainable development goals (SDG), this resulted in them being left out of policies and subsequently underfinanced. It remains unclear why stillbirths seem to be an invisible misfortune, despite the available evidence proving the magnitude of the problem. In the past decade, significant amount of research focused on stillbirths were implemented [4]. Nevertheless, there is consensus that stillbirths are understudied and under reported [13-15]. It has also been reported that stillbirth data from less developed countries are grossly incomplete making them unreliable and subsequently leading to inconclusive evidence [16].

There is limited information on risk factors for stillbirth in Namibia. This is one of the few studies done on stillbirths in Namibia and possibly the first to explore the risk factors of stillbirth in Otjozondjupa Region, which had the highest burden of stillbirth in the country in 2016 [17]. Therefore, the objectives of this study were to assess the maternal socio-demographic, maternal medical and obstetric risk factors associated with stillbirth, as well as to determine fetal related risk factors for stillbirth in Otjozondjupa Region, Namibia.

## Methods

**Study design:** an unmatched case control study was carried out at a case control ratio of 1:2.

**Setting:** this study was carried out in Otjozondjupa Region, which is located centrally in Namibia. The region is one of the largest regions in Namibia. It is sparsely populated with a catchment population of 149153 people, estimated at 0.6% annual population growth (Namibia Statistics Agency, 2013) and 4.2% pregnant women/expected delivery. It has 24 state healthcare facilities of which 5 conduct births daily and the rest only conduct emergency births.

**Participants:** the study target population were residents who birthed at any state healthcare facility in Otjozondjupa Region between January and December 2016. Cases were defined as women residing in Otjozondjupa, who birthed stillborn at state healthcare facilities in Otjozondjupa Region between January and December 2016 whilst controls as women residing in Otjozondjupa, who birthed live babies at state healthcare facilities in Otjozondjupa Region during the same period.

**Variables:** the study included common variables that had been pointed out by literature including age, marital status, employment and tribe. Twin pregnancy has long been associated with stillbirth, hence, all mothers with twin pregnancy were left out of the study during the selection stage. This was done to control for confounding.

**Data sources:** a pilot tested standardized questionnaire was utilized for data extraction. The questionnaire that comprised of close-ended questions consisted of three sections. Section one was to determine the maternal socio-demographic information, section two on the maternal medical and obstetric information and section three on the fetal related information. Additional interviews were avoided to avoid stirring traumatic feelings of past events particularly for the cases. Information contained in the clinical records was also deemed sufficient to carry out the study. Permission to carry out the study was obtained from the National Health Research Unit of the Ministry of Health and Social Services (MoHSS), Namibia, after the researcher had attained ethical clearance. The usage of records meant that no consent from the patients was needed. Records that were grossly incomplete were excluded from the study. Given its association to stillbirth, twin pregnancy was also excluded from the study to minimize confounding.

**Bias:** due to the retrospective nature of the study, recall bias could have been a potential bias. However, this was avoided by using second hand data, rather than conducting face to face interviews. The latter was also avoided due to the sensitive nature of the topic. Selection bias was avoided by conducting random sampling method. To counter for potential confounders resulting from the unmatched nature of the study, multiple logistic regression was conducted during data analysis.

**Study size:** the sample size was calculated using stat cal in Epi info 7.2. It was calculated at 95% confidence interval, 80% power, 11.1% least prevalence exposure (unemployment), odds ratio of 2.5 and 1:2 case control ratio. A total sample of 285 was required for this study, 95 cases and 190 controls (Kesley). Subjects were chosen using simple random sampling method. Whereby, patient files were assigned sequential numbers. Random numbers were then generated in Microsoft Excel and files with corresponding random numbers chosen as part of the sample.

**Quantitative variables:** the ages of the participants were grouped and analyzed as follows: ≤19 years, 20-29 years and 30 years and older. Tribe/ethnicity was categorized into the most appearing tribes in the region with an inclusion of ‘others´ to cater for the minority. Fetal weight was grouped into ≤2500g or >2500g. Babies weighing less than or equal to 2500 grams were regarded as premature babies. Other variables analyzed as categorical variables were: employment and marital status. Binary answers in form of ‘yes’ or ‘no’ replies were applied to the following variables: alcohol consumption, cigarette smoking, ANC attendance, premature delivery as well as high risk pregnancy. Other binary variables assessed as fetal risk factors were congenital malformations, cord enlargement or prolapse and fetal distress.

**Statistical methods:** Epi info 7 was used to analyze data. In bivariate analysis, crude odd ratios (cOR) were generated at 95% confidence intervals (CI) to find factors significant for stillbirth. The significant factors at p-value of < 0.05 and also other factors which are known risk factors by literature but were not significant at the bivariate level were then run in a multivariate logistic regression model to determine the true risk factors by generating adjusted odds ratios (aOR) with 95% confidence intervals. Multiple logistic regressions were used to control the confounding effect of different variables while assessing the effect of each variable on the likelihood of stillbirth occurrence.

## Results

**Participants:** about a 1170 patient records were eligible for this study. To stick to the sample size of the study, two hundred and eighty-five files were drawn to participate in the study. Of those six were grossly incomplete and thus removed from the sample. Another six were then re-drawn to replace the incomplete ones.

**Descriptive data:** a total of 285 women who delivered in Otjozondjupa Region from January 2016 to December 2016 participated in this study; 95 stillborn mothers and 190 live born mothers. The mothers in the 20 - 29 years age group had the highest frequency of 50.53% (n=144) whilst teenage mothers accounted for the lowest frequency 10.53% (n=30). This trend continued unabated when cases were compared to controls with the 20 - 29 age group scooping 50.53% (n= 48) in cases and 50.53% (n= 96) in controls whereas the teenage group had a low 6.31% (n=6) for cases and 12.63% (n=30) for controls. The overall mean age was 27 years (6.8 SD); this was similar to the mean age for controls 27 years (6.7 SD) whilst the mean for cases was 28 years (6.8 SD). The youngest woman was 16 years old whereas the oldest 45 years old. Table 1 further presents the socio-demographic characteristics of the cases and controls.

**Table 1:** socio-demographic characteristics of cases and controls

Characteristics			
**Age (years)**	**Cases (%) N = 95**	**Controls (%) N = 190**	**Total (%) N = 285**
≤ 19	6 (6.31%)	24 (12.63%)	30 (10.53%)
20- 29	48 (50.53%)	96 (50.53%)	144 (50.53%)
30-45	41 (43.16%)	70 (36.84%)	111 (38.94%)
**District of residence**			
Grootfontein	23 (24.2%)	47 (24.7%)	70 (24.6%)
Okahandja	22 (23.2%)	40 (21.1%)	62 (21.8%)
Okakarara	14 (14.7%)	32 (16.8%)	46 (16.1%)
Otjiwarongo	36 (37.9%)	71 (37.4%)	107 (37.5%)
**Employment status**			
Employed	21 (22.1%)	38 (20.0%)	59 (20.7%)
Unemployed	74 (77.9%)	152 (80.0%)	226 (79.3%)
**Marital status**			
Single	79 (83.2%)	163 (85.8%)	242 (84.9%)
Married	16 (16.84%)	27 (14.2%)	43 (15.1%)
**Tribe/ethnicity**			
Wambo	24 (25.3%)	49 (25.8%)	73 (25.6%)
Others	13 (13.7%)	21 (11.1%)	34 (11.9%)
Herero	22 (23.2%)	48 (25.3%)	70 (24.6%)
Damara/Nama	28 (29.5%)	63 (33.2%)	91 (31.9%)
San	8 (8.4%)	9 (4.74%)	17 (5.9%)

**Outcome data:** various risk factors were analyzed for. Most participants were reported to be HIV negative, n=71 (74.74%) for cases and n=170 (89.47%) for controls. Only 13 (13.68%) cases reported unknown HIV status. ANC attendance was good among participants, n=85 (89.47%) and n=186 (97.89%) for controls. Fifty-eight cases (61.05%) have had premature deliveries and 10 (10.53%) reported previous stillbirth. While most cases (50.53%) delivered before or at 36 weeks gestational age, contrary was reported in controls whereby 98.42% delivered after 36 weeks. The rate of high risk pregnancy was reported to be at 27.89% for cases and 40.00% for controls. Twenty-two (37.93%) cases were reported to have labored longer than 13 hours, as opposed to 27 (17.88%) for controls.

**Main results:** the study tested the association between maternal ages, district of residence, employment status, tribe/ethnicity and marital status and stillbirth. None of these potential socio-demographic factors were statistically significant during bivariate analysis. As previously mentioned, factors that were not significant in bivariate analysis but by literature are known risk factors were run in the multivariate logistic regression model to determine the true risk factors. Employment status, marital status, alcohol consumption and cigarette smoking were some of those factors. None of them yielded significance in multivariate analysis model either. More on socio-demographic risk factors is further explained in Table 2.

**Table 2:** bivariate and multivariate analysis of the socio-demographic risk factors for stillbirth

Risk factor	Cases (%)	Controls (%)	cOR (95% CI)	P value	aOR (95% CI)	P value
				0.87		
**Age (years)**						
20-29	48 (50.53%)	96 (50.53%)	1			
≤19	6 (6.32%)	24 (12.63%)	2.00 (0.76 - 5.22)			
≥30	41 (43.16%)	70 (36.84%)	0.85 (0.50 - 1.43)			
**Employment status**				**0.67**		**0.83**
Employed	21 (22.11%)	38 (20.00%)	1		1	
Unemployed	74 (77.89%)	152 (80.00%)	1.13 (0.62 - 2.07)		0.93 (0.50 - 1.74)	
**Tribe/ethnicity**				**0.69**		**0.63**
Wambo	24 (25.26%)	49 (25.79%)	1			
Damara/Nama	28 (29.47%)	63 (33.16%)	1.10 (0.56 - 2.13)			
Herero	22 (23.16%)	48 (25.26%)	1.06 (0.52 - 2.15)			
San	8 (8.42%)	9 (4.74%)	0.55 (0.18 - 1.60)			
Others	13 (13.68%)	21 (11.05%)	0.79 (0.33 - 1.84)			
**Marital status**				**0.55**		**0.95**
Married	16 (16.84%)	27 (14.21%)	1		1.02 (0.48 - 2.14)	
Single	79 (83.16%)	163 (85.79%)	0.81 (0.41 - 1.60)		1	
**Alcohol consumption**				**0.16**		**0.20**
No	78 (84.78%)	169 (90.37%)	1		1	
Yes	14 (15.22%)	18 (9.63%)	0.59 (0.28 - 1.25)		0.83 (0.34 - 2.00)	
**Cigarette Smoking**				**0.11**		**0.76**
No	83 (90.22%)	178 (95.19%)	1		1	
Yes	9 (9.78%)	9 (4.81%)	0.46 (0.17 - 1.21)		0.84 (0.28 - 2.50)	

Numerous maternal risk factors were found to be significantly associated with stillbirth. A significant association was made between HIV status and stillbirth in bivariate analysis (P < 0.001); this association however had no significance in multivariate analysis (P=0.72). Similar results were observed in previous stillbirth and type of delivery whose significant association also changed to insignificant during multivariate analysis (p=0.08 and 0.56). A total of 26.67% (n=76) women had unknown duration of labor due to various reasons including born before arrival (BBA) and elective caesarean section (CS). However, when these 76 with unrecorded duration of labor were excluded, the association between stillbirth and duration of labor was significant in multivariate analysis (aOR 4.04, 95% CI 1.56, 10.43, P=0.003). Women who labored for more than 13 hours had lower risk of birthing stillborn compared to those who labored 13 hours or less. High-risk pregnancy was also found to have a significant association to stillbirth (aOR 3.59 95% CI 1.35, 9.55, P=0.01). Gestational age and premature delivery too had significant associations with stillbirth. Women who delivered at term had higher risks of delivering stillborn, however, this association changed during multivariate analysis becoming protective against stillbirth (aOR 0.13, 95% CI 0.05, 0.33, P<0.001). Similarly, the association of gestational age changed from higher risk amongst women delivering after 36 weeks to lower risk (aOR 0.04, 95% CI 0.00, 0.25, P<0.001). Women without history of previous stillbirth appeared to have high risks of stillbirth in bivariate analysis (cOR 7.33, 95% CI 1.96, 27.32). Nevertheless, the association became insignificant in multivariate analysis (aOR 3.97, 95% CI 0.81, 14.43, P=0.08). Women with no history of ANC attendance had lower risk of stillbirth in multivariate analysis (aOR 0.07, 95% CI 0.00, 0.79, P=0.03). No association was found between BBA, pre-eclampsia and parity and stillbirth (P=0.10, 0.23 and 0.62). Due to incomplete data, the study was unable to asses for body mass index (BMI) as none of the participants had recorded height and only 2.1% (n=6) had recorded weight. More information on the examined risk factors is detailed in Table 3.

**Table 3:** bivariate and multivariate analysis of the maternal medical and obstetric related risk factors for stillbirth

Risk factor	Cases (%)	Controls (%)	cOR (95% CI)	P value	aOR (95% CI)	P value
**HIV status**				0.0001*		**0.723**
Positive	11 (11.58%)	15 (7.89%)				
Negative	71 (74.74%)	170 (89.47%)				
Unknown	13 (13.68%)	5 (2.63%)				
**ANC attendance**				0.001*		0.032*
Yes	85 (89.47%)	186 (97.89%)	1		1	
No	10 (10.52%)	4 (2.11%)	0.18		0.07 (0.00 - 0.79)	
**Premature delivery**				0.0001*		0.0001*
Yes	58 (61.05%)	13 (6.84%)	1		1	
No	35 (33.96%)	177 (93.16%)	16.38 (8.63 - 31.06)		0.13 (0.05 - 0.33)	
**Previous stillbirth**				0.0001*		**0.088**
Yes	10 (10.53%)	3 (1.58%)	1		1	
No	85 (89.47%)	187 (89.47%)	7.33 (1.96 - 27.32)		3.97 (0.81 - 14.43)	
**Gestational age (weeks)**				0.0001*		0.0001*
≤ 36	48 (50.53%)	3 (1.58%)	1		1	
>36	47 (49.47%)	187 (98.42%)	63.6 (18.99 - 213.37)		0.04 (0.00 - 0.25)	
**High risk pregnancy**				0.038*		0.010*
Yes	38 (27.89%)	53 (40.00%)	1		1	
No	57 (60.00%)	137 (72.11%)	1.72 (1.02 - 2.89)		3.59 (1.35 - 9.55)	
**Duration of labor (hours)**				0.0002*		0.0003*
≤ 13	36 (62.07%)	124 (82.12%)	2.80 (1.43 - 5.50)		4.04 (1.56 - 10.43)	
> 13	22 (37.93%)	27 (17.88%)	1		1	

* : P value statistically significant at <0.05, cOR: crude odds ratio; aOR: adjusted odds ratio

Congenital malformations and birth weight were found to be significant factors for stillbirth. Nonetheless, the association between congenital malformations and stillbirth dissolved during multivariate analysis (P=0.96). On the other hand, the association between birth weight and stillbirth remained statistically significant. Weight lower than 2500 grams have been found to have a higher risk of stillbirth than weight higher than 2500 grams (aOR16.58, 95% CI 8.71, 31.55, P <0.001). No association was made between cord enlargement, cord prolapse and fetal distress and stillbirth. Table 4 digests the results further.

**Table 4:** bivariate and multivariate analysis of the fetal related risk factors for stillbirth

Risk factor	Cases (%)	Controls (%)	cOR (95% CI)	P value	aOR (95% CI)	P value
**Congenital malformations**				0.044*		**0.964**
Yes	2 (2.11%)	0 (0.00%)				
No	93 (97.89%)	190 (100.00%)				
**Cord enlargement**				**0.156**		
Yes	1 (1.05%)	0 (0.00%)				
No	94 (98.95%)	190 (100.00%)				
**Cord prolapse**				**0.075**		**0.115**
Yes	3 (3.16%)	1 (0.53%)	1		1	
No	92 (96.84%)	189 (99.47%)	6.16 (0.63 - 60.06)		6.23 (0.63 - 60.73)	
**Fetal distress**				**0.218**		
Yes	2 (2.11%)	1 (0.53%)	1			
No	93 (97.89%)	189 (99.47%)	4.06 (0.36 - 45.40)			
**Birth weight (g)**				0.0001*		0.0001*
≤ 2500	60 (63.16%)	18 (9.47%)	1		16.58 (8.71 - 31.55)	
> 2500	35 (36.84%)	172 (90.53%)	16.38 (8.63 – 31.06)		1	

* :P value statistically significant at <0.05; cOR: crude odds ratio; aOR: adjusted odds ratio

## Discussion

This unmatched case-control study aimed at elucidating the risk factors associated with stillbirth. This study demonstrated that none of the examined socio-demographic factors had significant association. Though none of the factors were significantly associated, various socio-demographic factors have been associated by other studies. Maternal age for example has been termed a factor by studies done in Australia, Nepal, and Tanzania, which found older women to have a higher risk of delivering stillborn [18-20]. In contradiction, a study from Ghana reported that, women younger than 24 were found to have a greater risk of having stillborn [8]. Younger and older mothers may be more likely to have stillbirth due to various reasons including developing bodies, poor cooperation during delivery and reduced immunity respectively. In the present study, several medical and obstetric factors yielded a significant association. The importance of attending ANC services has continuously been emphasized. Despite the aforementioned, our results linked lack of ANC to lower risk of stillbirth. Several studies are in agreement that lack of ANC increases the risk of stillbirth in opposition to our findings [21,22]. Stillbirth is an important measure of the quality of ANC [23]. Our findings may be an indicator of poor quality of ANC in the region. The current study found that premature labor increases the risk of stillbirth, this was also supported by a study from Bangladesh [24]. Quite contrary, Korde-Nayak [25] found lower risk of stillbirth to women who delivered prematurely. The same results were observed in Ghana [8]. Perhaps the increased association between premature labor and stillbirth demonstrated in our study is due to the lack of aggressive emergency and obstetric care the women receive upon presenting at health facilities. It may also be due to limited resources and lack of specialists in the region.

Longer duration of labor has frequently been associated with increased risk of stillbirth [21,24,26]. Different studies have analyzed the duration of labor range differently. Omo-Aghoja *et al*. (2014) reported that in Nigeria labor lasting more than 8 hours placed the baby at risk. The same was reported by Badimsuguru *et al*. (2016) in Ghana, and by Jammeh *et al*. (2010) in Burkina Faso, where labor duration was longer than 12 hours and 1 day, respectively [8,21,22]. In disagreement with the above findings, our current study presented that, mothers who labored longer than 13 hours had lower risks of stillbirth. Gestational age of the pregnancy at delivery does play a role in the outcome of the pregnancy. Our findings showed a significant association between gestational age and stillbirth. Whereby women who labored before 36 weeks had elevated risk of stillbirth than those who labored after that. Association between gestational age and stillbirth is nothing new as several studies have found association over the years [27-29]. A study by Rosenstein *et al*. [29] in California found that the risk of stillbirth increase with an increase in gestational age. It however, reported that the highest risk occur when the pregnancy is postdated, a finding that was supported by another study done by Shyam [27] in India. Low birth weight was the only fetal risk factor to which an association was made. We found that birth weight of ≤ 2500 g increase the risk of stillbirth. Studies by Joseph *et al*. [30] and Lawn *et al*. [4] underlined that low birth weight babies are likely to die in their first week of life due to under-development, hypothermia and hypoglaecemia [30]. This was similarly supported by Ashish *et al*. [18] and Omo-Aghoja *et al*. [21].

The study suffered some limitations. The study only looked at women who delivered at state healthcare facilities. This means women who delivered at home and at private healthcare facilities were not included in the study. This means the results obtained may not be a true reflection of the entire region. This also means that the results of this study cannot be generalized to all women in the region neither to other regions in the country since the study was only conducted within Otjozondjupa. Another major limitation was incomplete records. Since only grossly incomplete records/files were excluded in the study, some files included were still incomplete to a certain degree. This made it impossible to analyze for some factors. The researchers recommend that future studies are supplemented by interviews to account for missing information.

## Conclusion

Stillbirth remains a worrisome matter in Otjozondjupa Region. No socio-demographic factor was associated to still birth by this study. Maternal medical and obstetric factors associated with stillbirth were: ANC attendance, premature delivery, gestational age, high-risk pregnancy and duration of labor. ANC attendance did not improve the birth outcome. This may mean ANC services in Otjozondjupa needs strengthening so risk factors are diagnosed early and handled appropriately. Low birth weight was the only factor associated with stillbirth. Continuous aggressive intrapartum care may be the answer to women in preterm labor to avert stillbirths amongst preterm babies. More studies in different settings in Namibia are encouraged to inform decisions aimed at reduction of perinatal mortality.

### What is known about this topic

- *Stillbirth is a major concern in sub-Saharan Africa*;
- *Some risk factors are common and cuts across board*.

### What this study adds

- *Stillbirth is a problem in Otjozondjupa Region, the region needs to strengthen the quality of ANC such that risk factors are detected early and effectively responded to, this way, ANC can improve birth outcome;*
- *Some risk factors associated to stillbirth by this study have also been associated with stillbirth in other countries; this means the study recommendations may be utilized by the Namibian Ministry of Health to improve ante and perinatal services in the country*.
